# Spatial-temporal analysis of breast cancer in upper Cape Cod, Massachusetts

**DOI:** 10.1186/1476-072X-7-46

**Published:** 2008-08-13

**Authors:** Verónica M Vieira, Thomas F Webster, Janice M Weinberg, Ann Aschengrau

**Affiliations:** 1 Department of Environmental Health, Boston University School of Public Health, Talbot 4W, 715 Albany Street, Boston, MA 02118, USA; 2 Department of Biostatistics, Boston University School of Public Health, Crosstown, 715 Albany Street, Boston, MA 02118, USA; 3 Department of Epidemiology, Boston University School of Public Health, Talbot 3E, 715 Albany Street, Boston, MA 02118, USA

## Abstract

**Introduction:**

The reasons for elevated breast cancer rates in the upper Cape Cod area of Massachusetts remain unknown despite several epidemiological studies that investigated possible environmental risk factors. Data from two of these population-based case-control studies provide geocoded residential histories and information on confounders, creating an invaluable dataset for spatial-temporal analysis of participants' residency over five decades.

**Methods:**

The combination of statistical modeling and mapping is a powerful tool for visualizing disease risk in a spatial-temporal analysis. Advances in geographic information systems (GIS) enable spatial analytic techniques in public health studies previously not feasible. Generalized additive models (GAMs) are an effective approach for modeling spatial and temporal distributions of data, combining a number of desirable features including smoothing of geographical location, residency duration, or calendar years; the ability to estimate odds ratios (ORs) while adjusting for confounders; selection of optimum degree of smoothing (span size); hypothesis testing; and use of standard software.

We conducted a spatial-temporal analysis of breast cancer case-control data using GAMs and GIS to determine the association between participants' residential history during 1947–1993 and the risk of breast cancer diagnosis during 1983–1993. We considered geographic location alone in a two-dimensional space-only analysis. Calendar year, represented by the earliest year a participant lived in the study area, and residency duration in the study area were modeled individually in one-dimensional time-only analyses, and together in a two-dimensional time-only analysis. We also analyzed space and time together by applying a two-dimensional GAM for location to datasets of overlapping calendar years. The resulting series of maps created a movie which allowed us to visualize changes in magnitude, geographic size, and location of elevated breast cancer risk for the 40 years of residential history that was smoothed over space and time.

**Results:**

The space-only analysis showed statistically significant increased areas of breast cancer risk in the northern part of upper Cape Cod and decreased areas of breast cancer risk in the southern part (p-value = 0.04; ORs: 0.90–1.40). There was also a significant association between breast cancer risk and calendar year (p-value = 0.05; ORs: 0.53–1.38), with earlier calendar years resulting in higher risk. The results of the one-dimensional analysis of residency duration and the two-dimensional analysis of calendar year and duration showed that the risk of breast cancer increased with increasing residency duration, but results were not statistically significant. When we considered space and time together, the maps showed a large area of statistically significant elevated risk for breast cancer near the Massachusetts Military Reservation (p-value range:0.02–0.05; ORs range: 0.25–2.5). This increased risk began with residences in the late 1940s and remained consistent in size and location through the late 1950s.

**Conclusion:**

Spatial-temporal analysis of the breast cancer data may help identify new exposure hypotheses that warrant future epidemiologic investigations with detailed exposure models. Our methods allow us to visualize breast cancer risk, adjust for known confounders including age at diagnosis or index year, family history of breast cancer, parity and age at first live- or stillbirth, and test for the statistical significance of location and time. Despite the advantages of GAMs, analyses are for exploratory purposes and there are still methodological issues that warrant further research. This paper illustrates that GAM methods are a suitable alternative to widely-used cluster detection methods and may be preferable when residential histories from existing epidemiological studies are available.

## Background

Surveillance of routinely collected data for unusual clusters of disease in space and time is a topic of general importance. Many epidemiologists resist community pressures to conduct cluster investigations believing they rarely provide conclusive information regarding the etiology of the disease. This is because cluster investigations often combine unrelated diseases; contain too few cases to be meaningful; have "gerrymandered" boundaries; and examine only cases without taking into account differences in population density [1]. Even studies of registry data ignore many known risk factors and latency. Maps that ignore latency may be flatter if population movement is random with respect to disease status [2]. Nevertheless, cluster investigations are an important part of responding to public concerns, even if no new etiologic knowledge is gained [3,4].

**Figure 1:**
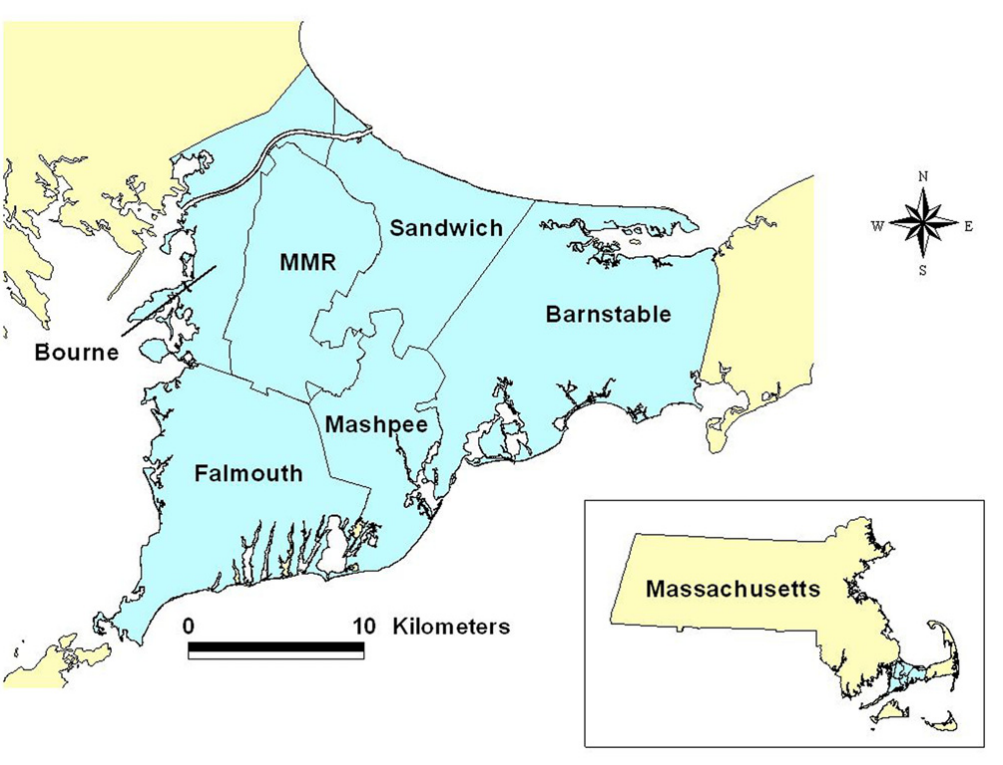
**Geographic location of the upper Cape Cod study area**. Upper Cape Cod consists of the five towns: Barnstable, Bourne, Falmouth, Mashpee, and Sandwich. Map was reproduced with permission from Environmental Health Perspectives (Paulu et al. 2002).

Community concern over elevated cancer rates in upper Cape Cod, Massachusetts, USA (Figure 1) prompted several epidemiological studies that investigated possible environmental risk factors, including air and water pollution associated with the Massachusetts Military Reservation (MMR), pesticide applications to cranberry bogs, particulate air pollution from a large electric power plant, and tetrachloroethylene-contaminated drinking water from vinyl-lined asbestos cement distribution pipes [5-16]. Some positive associations were observed, but researchers concluded that environmental exposures they investigated could only explain a portion of the excess cancer incidence.

We combined data from two of these existing population-based case-control studies of breast cancer in upper Cape Cod [6,10] to investigate the associations between space, time, and breast cancer risk. The detailed information on individual-level covariates and residential histories beginning in 1947 makes these existing studies a useful data set for spatial-temporal analysis. Cases were identified using cancer registries while controls provided an estimate of the underlying population density. Participants or next-of-kin were interviewed to obtain relevant data on covariates and residential history. Information collected in the interview included age at diagnosis or index year, family history of breast cancer, personal history of breast cancer (before current diagnosis or index year), age at first live birth or stillbirth, occupational exposure to solvents, history of benign breast cancer, race, body mass index, history of radiation exposure, alcohol use, history of cigarette smoking, past use of diethylstilbestrol (DES), oral contraceptives and menopausal hormones, marital status, religion, education level, exposure to tetrachloroethylene from drinking water distribution pipes, and physical activity level. The residential history was geocoded using geographical information systems (GIS) to produce a point-based data set. Generalized additive models (GAMs), a type of statistical model that combines smoothing with the ability to analyze binary outcome data and adjust for covariates, provide a useful framework for spatial analysis of population-based case-control data [17-22]. GAMs allow for smoothing of data while simultaneously adjusting for known risk factors.

An import consideration in spatial-temporal analyses is how to define time. Many space-time cluster analyses examine location at time of disease diagnosis [23]. For a disease with a long latency like breast cancer, the time of etiologic interest is not when the disease was diagnosed but rather when the exposure occurred. Our prior spatial analyses [19-22] considered time in terms of latency by restricting inclusion in the analysis to the residences occupied by participants at least twenty years prior to the diagnosis (for cases) or index year (for controls).

Although latency is a more relevant time measure for breast cancer than diagnosis year, it does not address timing in relation to exposure occurrence. For example, in a 20-year latency analysis, two participants who moved into the same neighborhood in 1970 would not both be in the analysis if one was diagnosed in 1983 (13-year latency) and the other was diagnosed in 1993 (23-year latency). If an environmental exposure occurred in 1970, then a fixed latency analysis may not predict the correct breast cancer risk. Calendar years of residency are important because the magnitude of exposure can vary over time, and past rather than current exposures may be more relevant for breast cancer etiology. Residency duration is also relevant to etiologic exposures assuming duration of exposure is related to duration of residency. A participant who lived at a residence near the source of an environmental contaminant for five years but moved before the contamination occurred would be unexposed, while someone who lived in the same residence for five years after the contamination occurred would be exposed. Likewise, a person who lived at an exposed residence for 5 years may have a different disease outcome than someone who lived there for 35 years. Our present work measures time both in calendar years of residency and residency duration.

In this paper, we examine breast cancer risk with a space-only analysis where time is not considered, a time-only analysis where space is not considered, and a spatial-temporal analysis that allows both time and space to vary. We used continuous residential histories so participants who moved away from the study area and later returned were excluded. We report global statistics for disease clustering and visualize breast cancer risk using GIS.

## Results

### Spatial analyses

**Figure 2:**
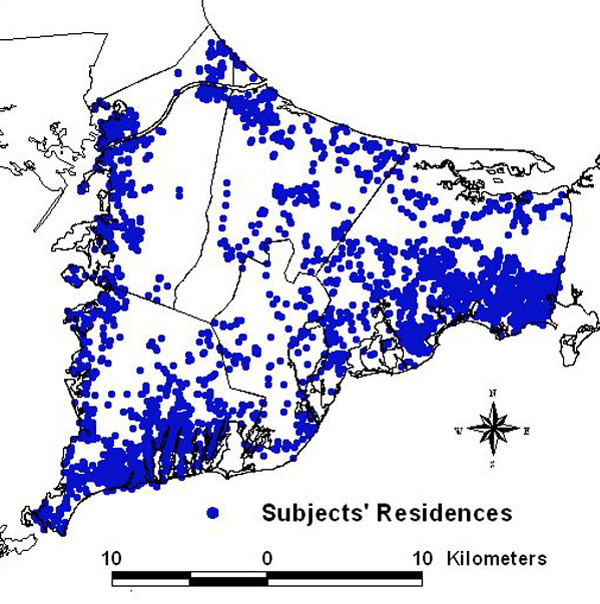
**Spatial distribution of breast cancer participants over the complete residential history period (1947–1993)**. Points represent the residences of the participants. Locations have been geographically altered to preserve confidentiality. Actual locations were used in the analyses.

We investigated the association between residential history since 1947 and risk of breast cancer during 1983–1993 using data from two population-based case-control studies [6,10]. There were a total of 1,631 participants with continuous residential histories in the study area. Because participants moved within the study area, they contributed a total of 2,477 residences to the spatial analysis (Table 1). Over 35% of the participants moved at least once within the study area during the residential history period (Table 2). Figure 2 shows the spatial distribution of participants' residences over their entire residential history in the study area. To preserve confidentiality, the figure was created by randomly placing residences within a 1.2 km grid that includes the actual location. Actual locations were used in the analysis.

**Table 1:** Years of diagnosis and residential history characteristics of study participants. The analyses used data from two existing population-based case-control studies.

**Characteristic**	**Study 1**	**Study 2**	**Final Dataset**
**Diagnosis/Index Years**	1983–86	1987–93	1983–93
**No. of Cases**	207	453	660
**No. of Case Residences**	327	684	1,011
**Mean No. of Residences per Case**	1.58	1.51	1.53
**No. of Controls**	526	445	971
**No. of Control Residences**	762	704	1,466
**Mean No. of Residences per Control**	1.45	1.58	1.51
**Case/Control Ratio**	0.39	1.02	0.68
**Total No. of Participants**	733	898	1,631
**Total No. of Residences**	1,089	1,388	2,477
**Mean No. of Residences per Participants**	1.49	1.55	1.52

**Table 2:** Number of upper Cape Cod residences by participant status

**Number of Residences**	**Cases**	**Controls**	**Total Participants**
1	551	810	1,361
2	70	101	171
3	27	39	66
4	5	18	23
5	5	2	7
6	1	0	1
7	1	1	2

Total	660	971	1,631

**Figure 3:**
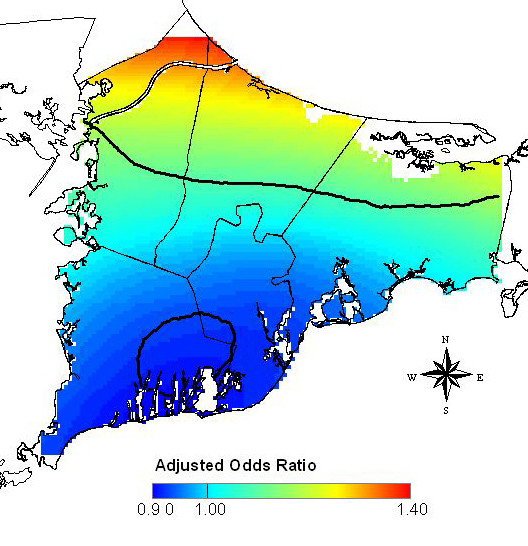
**Spatial analysis of breast cancer risk during 1983–1993**. The map shows statistically significant increased ORs in the north and decreased ORs in the south (global p-value = 0.04). ORs are relative to the whole study area based on participants' residences in upper Cape Cod over the residential history period from 1947 to 1993.

The space-only analysis included all eligible addresses (n = 2,477) in the residential history. The optimal span for the adjusted space-only GAM was 95%. The model was adjusted for the time period of case ascertainment (i.e., study 1 or study 2), age at diagnosis or index year, year of diagnosis or index year, vital status at interview, family history of breast cancer, personal history of breast cancer (before diagnosis or index year), parity and age at first live- or stillbirth, history of radiation exposure, and race. The large span size indicates the data are close to planar, but the plane was tilted with increased odds ratios (ORs) in the north of the study area and decreased ORs in the south (Figure 3). Predicted ORs ranged from 0.90 to 1.40. The global permutation test for the null hypothesis that case status does not depend on location (i.e., a flat surface with no slope) resulted in a p-value of 0.04, indicating that there was a significant association between location and breast cancer risk during 1983–1993. Figure 3 also shows the resulting 2.5% and 97.5% contours of the pointwise permutation tests.

### Temporal analyses

Combining data from two population-based case-control studies resulted in a residential history that spanned 47 years (1947–1993, Table 1). Over 15% (n = 254) of the participants were already living in the study area at the start of the residential history (1947). The remaining 1,377 participants moved into the study area after 1947. Of those 254 residents living in the study area at the start of the residential history, 99 were cases and 155 were controls, for a case/control ratio of 0.64. The case/control ratio for the entire analytic population is 0.68 (Table 1).

We included all eligible participants in the time-only analyses (n = 1,631) and adjusted for the time period of case ascertainment (i.e., study 1 or study 2), age at diagnosis or index year, year of diagnosis or index year, vital status at interview, family history of breast cancer, personal history of breast cancer (before diagnosis or index year), parity and age at first live- or stillbirth, history of radiation exposure, and race.

We first analyzed time using a one-dimensional smooth of the participants' residency durations in the study area. Residency durations, calculated as the difference between diagnosis/index year and earliest year in the study area, ranged from 1 to 47 years. The earliest year was either the year a participant moved to upper Cape Cod or 1947 for participants already living there. Half of the participants had residency durations of less than 15 years. Another 18% of the participants (n = 297) had durations over 35 years. The majority of these participants (n = 254) were already living in the study area in 1947, the beginning of the residential history.

**Figure 4:**
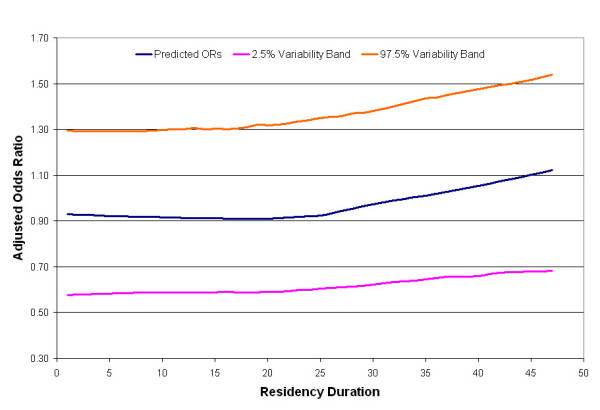
**One-dimensional temporal analysis of residency duration in study area**. The risk of breast cancer during 1983–1993 increases steadily after 25 years, but the association is not statistically significant (global p-value = 0.49).

The optimal span for the univariate smooth term in the residency duration model was 95% of the data. Figure 4 shows that the risk of being diagnosed with breast cancer during 1983–1993 begins to increase with 25 years of residency duration (blue line). Predicted ORs ranged from 0.91 to 1.12. The global permutation test for the null hypothesis that case status does not depend on duration had a p-value of 0.49, indicating that there was no significant association between duration and breast cancer risk during 1983–1993. Figure 4 also shows the resulting 2.5% (purple line) and 97.5% (orange line) variability bands.

We next examined time using a one-dimensional smooth of calendar year. We examined the earliest calendar year a participant lived in the study area rather than diagnosis year because it is potentially more relevant for breast cancer etiology. Because the case ascertainment periods for the first and second studies were 1983–1986 and 1987–1993, respectively, only participants of the second study had the opportunity to move to the study area during the early 1990s. This accounts for the sharp drop in the number of participants moving to the study area between the mid 1980s and the early 1990s. The median move year was 1976.

**Figure 5:**
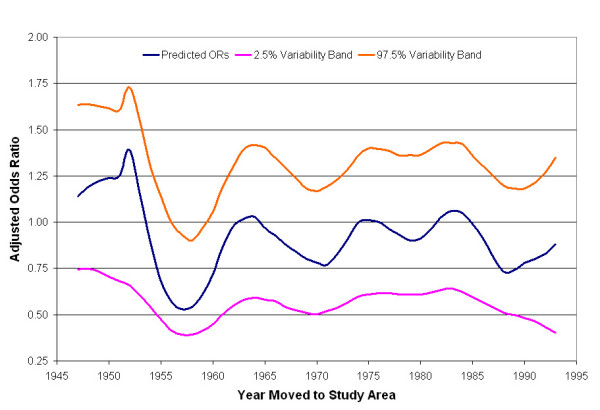
**One-dimensional temporal analysis of earliest calendar year in study area**. Results suggest earlier years (1947–1952) are associated with higher breast cancer risk during 1983–1993 (global p-value = 0.05).

The optimal span for the univariate smooth term in the calendar year model was 25% of the data. Figure 5 shows that the risk of being diagnosed with breast cancer during 1983–1993 was elevated for the years 1947–1952, but then decreased and remained low until 1964 when it became more level (blue line). Predicted ORs for the entire time period ranged from 0.53 to 1.38. The global permutation test for the null hypothesis that case status does not depend on the earliest year a participant lived in the study area produced a p-value of 0.05. Figure 5 also shows the resulting 2.5% (purple line) and 97.5% (orange line) variability bands. In general, the results of the one-dimensional time-only analyses suggest that longer durations and earlier residency years may increase breast cancer risk during 1983–1993, although residency duration was not significantly associated with breast cancer risk.

**Figure 6:**
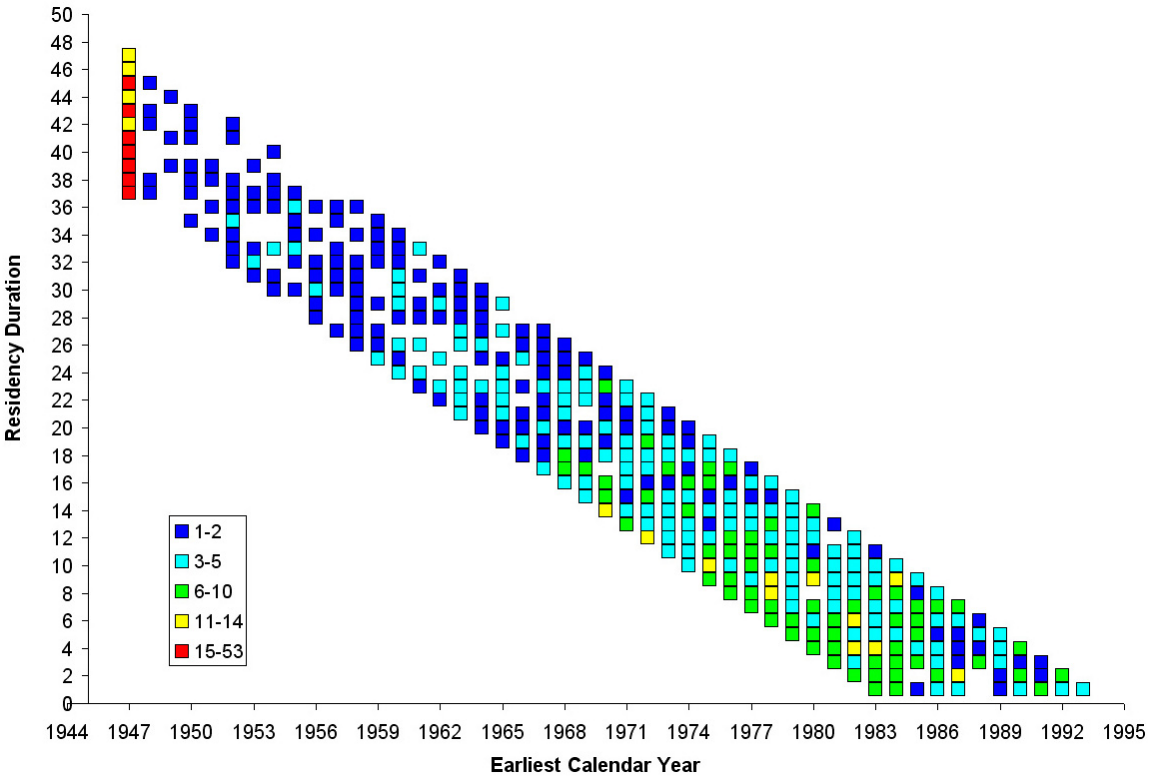
**Frequency and distribution of the study participants by earliest calendar year lived in study area and residency duration**. Colored squares indicate the number of participants with various combinations of earliest calendar year and residency duration. The earliest year is either 1947 for participants living in the study area at the start of the residential history, or the year participants moved to the study area. Residency duration is calculated as the difference between earliest year and diagnosis year for cases or index year for controls.

Figure 6 shows the frequency and distribution of study participants by earliest calendar year they lived in upper Cape Cod and residency duration. Colored squares indicate the number of participants with various combinations of earliest year and residency duration. Again, because 15% of participants (n = 254) were already living in upper Cape Cod at the start of the residential history, there is a high concentration of participants with earliest year of 1947.

**Figure 7:**
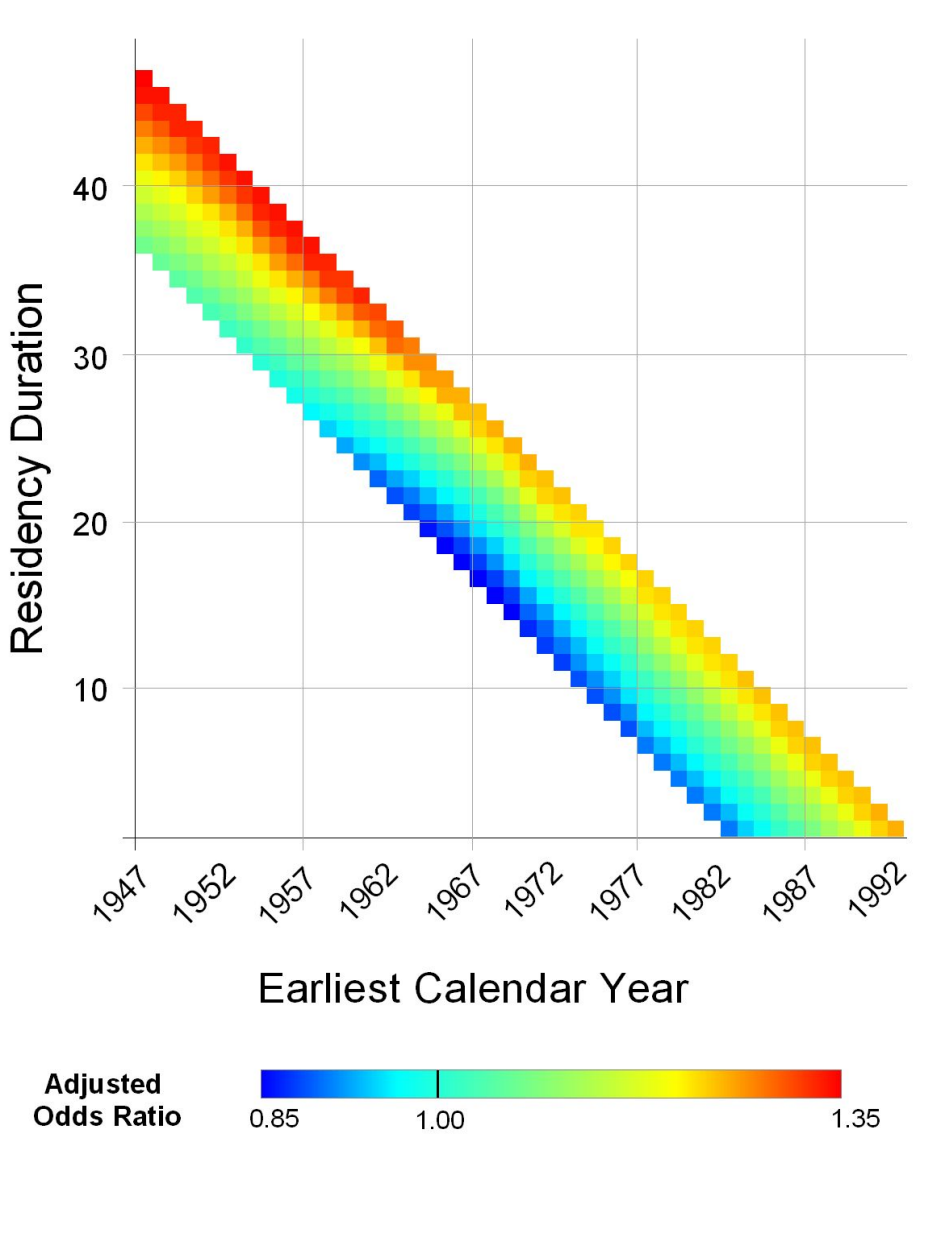
**Two-dimensional temporal analysis of breast cancer risk during 1983–1993**. The figure shows increased breast cancer risk with higher residency duration where time is represented in the model as a bivariate measure of earliest calendar year lived in the study area and residency duration. This association was not statistically significant (global p-value = 0.40).

We predicted the adjusted breast cancer odds ratios for every valid combination of earliest calendar year and residency duration. The optimal span for the bivariate smooth term was 95% of the data. Figure 7 shows that the risk of being diagnosed with breast cancer during 1983–1993 is increased for longer residency durations and decreased for shorter durations over all calendar years. Predicted ORs ranged from 0.90 to 1.20. The map was flat based on the global statistic (p = 0.40), indicating that duration and calendar year, independent of location within the study area, did not affect a participant's risk of being diagnosed with breast cancer during 1983–1993.

### Spatial-temporal analyses

We used GAMs and GIS to create a movie of continuous space-time animation for breast cancer risk during 1983–1993 based on the location and calendar years participants lived in upper Cape Cod. The model included a bivariate smooth of longitude and latitude similar to that of the space-only analysis, but the data were divided into overlapping datasets of 11-year time spans to smooth over time. The 11-year span size was chosen because the optimal span for the temporal analysis of calendar year was 25% of the data. The analysis started with years 1947–1957 representing the first frame of the movie (which corresponds to year 1947 on the movie timeline) and moved a year at a time until 1983–1993 (year 1983 on the movie timeline), for a total of 37 map frames. The span size for the smooth of longitude and latitude was 20%. This was the optimal span for the first 11-year dataset and was used for all the datasets. This ensures that the differences in maps are not due to differences in the span size.

Although residency duration was not explicitly included as a model term in this analysis, the longer a participant's duration, the more datasets to which the participant contributed. For example, a participant that moved to the study area in 1948 was included in all datasets. Consequently, participants with longer residency durations contributed more to the overall spatial-temporal analysis.

**Figure 8:**
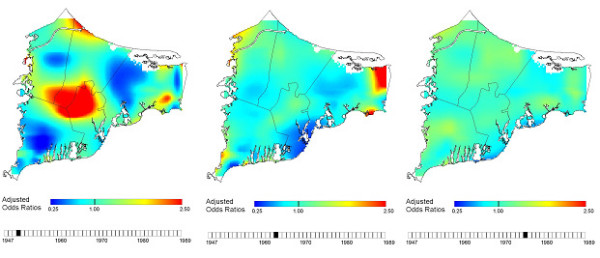
**Spatial-temporal analysis of breast cancer risk during 1983–1993**. Selected map frames show changing patterns of breast cancer risk during 1983–1993 based on participants' historical residences.

The spatial-temporal analyses found a large area of elevated breast cancer risk during 1983–1993 corresponding to historical residences in the center of the study area near the Massachusetts Military Reservation from 1947 to 1956. Odds ratios for these ten maps (1947–1956) ranged from 0.25 to 2.5, and global p-values ranged from 0.01 to 0.05, indicating a statistically significant association between residential histories during 1947–1956 and breast cancer risk during 1983–1993. A smaller area of elevated breast cancer risk during 1983–1993 was also seen corresponding to residences in the northeast region of upper Cape Cod during the 1960s, but the global p-values for maps of these years were not statistically significant. Figure 8 shows selected map frames from the movie. To view the entire movie video, see Additional file 1 or visit . It is important to emphasize that the movie visualizes the risk of being diagnosed with breast cancer during 1983–1993 that is associated with the location of participant residences in upper Cape Cod during historical time periods. It does not show the incidence of breast cancer during the historical periods.

## Discussion

Space-time maps allow for disease-pattern analysis of cases and controls using historical residences [24]. The GAM method visualizes breast cancer risk while adjusting for known confounders and testing for the statistical significance of location and time. Our analyses illustrate its application as an alternative to other widely-used cluster methods when residential histories from epidemiological studies are available. No one method is ideal for every cluster investigation and each contributes different and important features to space-time analyses. The Knox method [25,26] defines pairs of events as being either close or not close in time or space. This method does not require the large amounts of data and residential histories used by the GAM method. An advantage of the GAM method is that theoretical considerations of bias and variance are used to choose an optimal smoothing span [17], whereas the Knox method uses arbitrary cutoff points to determine clustering [27,28]. The K-function method proposed by Diggle et al. [29] improves upon this limitation by using a range of cutoffs. The smooth term in the GAM models also adapts to changes in population density [30], which is an important issue in our study area, where the population is concentrated along the coast. The Knox test, while appropriate in many cases, is not ideally suited for our study area because of potential bias stemming from uneven population shift across the geographic study area [31].

Kulldorff's space-time SaTScan method detects cancer clusters of specified shape and provides center coordinates and relative risk measure for the mostly likely cluster [32]. SaTScan is a widely-used and effective tool for cancer cluster analyses using registry-based data. The space-time SaTScan typically uses addresses at diagnosis to determine if cases are clustered, and so does not provide insight into timing of exposure and residential histories [33] that the GAM method provides in our current analyses. GAM methods predict cancer risk based on the entire residential history, not just one time point, as well as duration of residence. The latter is particularly important for diseases with long latency periods where exposure likely occurred many years prior to diagnosis.

Kwan et al. use an interesting three dimensional geovisualization method to display movement across time and space [33]. This method is an appropriate option for small datasets, but the network of lines used for visualization makes this method impractical for large epidemiological studies. Jacquez et al. have developed a useful test for cluster detection that accounts for large residential histories, can accommodate various interpretations of time, and identifies which events are clustered [34]. The GAM method does not identify clusters of events but instead identifies areas of increased risk on a continuous risk map. While visualization of residential history is useful for exploratory purposes, statistical analyses are needed to identify significant associations [35]. Our spatial-temporal analysis combines the visualization of odds ratios while allowing for hypothesis-testing to determine clusters of significantly increased or decreased risk.

Although GAMs have many advantages, a number of issues remain. The GAM analysis uses one constant optimal smoothing span for space and another for time. While this ensures that mapped results are unaffected by the degree of smoothing, we ideally would use smoothing spans determined to be optimal in a combined time-space framework. Further work is needed to resolve this methodological issue. GAMs may also exhibit edge effects, which are biased behavior at the edges of the data [31]. As much of our spatial data is found along the edges (i.e., population is denser by the coastline), this issue remains a concern despite our work with synthetic data showing little, if any, edge effect [20].

We identified areas with significantly increased or decreased risk using pointwise hypothesis tests. By making these multiple comparisons, we increase the likelihood of finding significant hot or cold spots by chance alone. Although we make no adjustment for multiplicity, we only conducted pointwise tests if the global deviance test indicated that the map was unlikely to be flat. The location of significant hot and cold spots should be considered exploratory.

There are also limitations implicit in using epidemiological data for secondary analysis. There are sparse data for the earlier calendar years of the temporal datasets, which affect the power of our clustering tests. To date, our spatial-temporal analysis methods also do not directly consider duration of time living at a residence, an important component to exposure. We are currently exploring additional GAM methods for simultaneously smoothing calendar year, duration, and location [35].

## Conclusion

Spatial-temporal analysis of the breast cancer data may help identify new exposure hypotheses that warrant future epidemiologic investigations with detailed exposure models. We performed a spatial-temporal analysis of breast cancer risk during 1983–1993 that combined statistical and visualization tools to examine time and place of participants' residences as proxies for unknown environmental exposures. Our results indicated only slight increases in breast cancer risk when we considered location or time alone while controlling for known risk factors. However, when we considered the combined effects of both space and calendar year of residency, we observed a strong statistically significant association between breast cancer risk and living near the Massachusetts Military Reservation from 1947 to 1956 (p-value range: 0.01 to 0.05; OR range: 0.25–2.50). These results suggest that further analyses be conducted to explore the reason for this geographic association. If the association is not a result of residual confounding or bias, then activities on the Military Reservation during that time window should be investigated to provide insight into possible exposure routes (i.e., ingestion of drinking water contaminated by improperly disposed chemicals; inhalation of air following recent mortar detonation). The current analyses illustrate the usefulness of GAMs and GIS to visualize cancer risk, adjust for known confounders, and test for the statistical significance of location and time. Our method is particularly useful residential histories are available.

## Methods

### Study population

We investigated the association between residential history and breast cancer in upper Cape Cod, Massachusetts (USA) using data from two population-based case-control studies [2,6]. Participants or their next-of-kin completed an extensive interview, providing information on demographic characteristics (age, sex, marital status, education), a forty-year residential history, and potential confounders such as smoking, family history of cancer, and occupational exposure to carcinogens. The residential histories ranged from 1943–1987 for the first study and 1947–1993 for the second study.

The Massachusetts Cancer Registry was used to identify incident cases of breast cancer. Cases were diagnosed from 1983–1986 for the first study and 1987–1993 for the second study. Participants were restricted to permanent residents of the upper Cape Cod region with complete residential histories. Table 1 shows the number of cases and controls by study period. In the first study, there were 207 cases with diagnosis year between 1983 and 1986 who contributed 327 upper Cape Cod residences between 1947 and 1986. In the second study, there were 453 cases with diagnosis year between 1987 and 1993 who contributed 684 upper Cape Cod residences between 1947 and 1993.

Controls were chosen to represent the underlying population that gave rise to the cases in a manner that was not spatially biased, that is, permanent residents of the study area during the case ascertainment period. Because many cases were elderly or deceased at diagnosis, three different sources of controls were used: (1) random digit dialing to identify living controls less than 65 years of age; (2) Centers for Medicare and Medicaid Services (formerly the Health Care Financing Administration) to identify living controls 65 years of age or older; and (3) death certificates to identify controls who had died from 1983 onward. See Aschengrau et al. [6,10] for a detailed description of the methods used to define the study population.

Controls were frequency matched to cases on age and vital status. "Index years" were randomly assigned to controls in a distribution similar to that of diagnosis years for cases. We used index years to estimate length and time of environmental exposure for controls in a fashion comparable to that of cases. Controls that moved to the study area after the assigned index year were excluded from the analyses. In the first study, there were 526 controls with index year between 1983 and 1986 who contributed 762 upper Cape Cod residences between 1943 and 1986. In the second study, there were 445 controls with index year between 1987 and 1993 who contributed 704 upper Cape Cod residences between 1947 and 1993 (see Table 1).

### Geographical information system (GIS)

Residential addresses reported by participants in the upper Cape Cod area from 1947 to the diagnosis or index year were eligible for analysis. We chose 1947 because it was the first year shared by both studies' residential histories. We excluded all addresses where residency ended before 1947 or began after diagnosis/index year, and participants who moved away and later returned to the study area. The combined breast cancer data set included 660 cases with 1,011 residential locations and 971 controls with 1,466 locations (see Table 1).

Locations of the participant residences were geocoded using the Massachusetts State Plane Coordinate System with North American Datum 1983 (NAD1983) and linked to the participant's interview data. Geocoding was done without knowledge of case/control status, and the final data were checked for accuracy.

### Generalized additive modeling (GAMs)

We used generalized additive models (GAMs) to examine breast cancer risk for 1983–1993 in (1) a spatial analysis of residence location that did not consider time, (2) a temporal analysis of calendar year and residency duration that did not consider space, and (3) an analysis that combined both space and time. Given the dependency of etiologically meaningful exposure on both duration and calendar year, we analyzed these two measures of time both individually and in a combined analysis. All analyses were adjusted for the time period of case ascertainment (i.e., study 1 or study 2), age at diagnosis or index year, year of diagnosis or index year, vital status at interview, family history of breast cancer, personal history of breast cancer (before diagnosis or index year), parity and age at first live- or stillbirth, history of radiation exposure, and race. Other covariates including education and usual adult body mass index (BMI) were examined but did not change the appearance of the maps. Women with missing covariate data or non-continuous residency in the study area were excluded from the analysis.

We estimated local disease odds using GAMs, a form of non-parametric/semi-parametric regression with the ability to analyze binary outcome data while adjusting for covariates [17]. We use either a univariate S(x_1_) or bivariate smooth S(x_1_, x_2_)

(1) logit [p()] = S() + **γ'z**

where the left-hand side is the log of the disease odds, S is the univariate or bivariate smooth term, **z **is a vector of covariates, and **γ **is a vector of parameters. Univariate smooths were used in the individual models for earliest year lived in the study area and residency duration; bivariate smooths were used in the analyses for space (longitude (x_1_) and latitude (x_2_)) and calendar year/duration (earliest year (x_1_) and duration (x_2_)). We used a loess smooth which adapts to changes in data density [17]. The amount of smoothing performed by loess depends on the size of the neighborhood of points. In general, small neighborhoods reduce bias but increase variance. Conversely, larger neighborhoods produce smoother surfaces resulting in increased bias and reduced variability. As the neighborhood increases in size, more data points receive non-zero weights and the loess smoother approaches a linear regression. Theoretical considerations use the bias and variance to provide several methods for choosing an optimal neighborhood size, also called bandwidth or span [17]. We determined the optimal amount of smoothing for the space-only and time-only analyses by minimizing the Akaike's Information Criterion (AIC). The AIC approximates the deviance-based cross validation using the average deviance of a model penalized by the number of degrees of freedom. Both local and global minima of the AIC can exist. To find a global minimum, we plot the AIC curve for a large range of span sizes. For the space-time analysis, we used the optimal span of the time-only analysis. We converted from log odds to odds ratios (ORs) using the whole study population as the reference, dividing the predicted odds by the odds calculated by the reduced model while omitting the smooth term.

GAMs also provide a framework for hypothesis testing. We first tested the null hypothesis that case status does not depend on the smooth term using the difference of the deviances of model (1) with and without the smooth term. We estimated the distribution of the global statistic under the null hypothesis using a permutation test. We condition on the number of cases and controls, preserving the relationship between case/control status and covariates, and randomly assign individuals to locations. We carry out 999 permutations of location in addition to the original. For each permutation, we run the GAM using the optimal span of the original data and compute the deviance statistic. We divide the rank of the observed value by 1000 to obtain a p-value. We used a p-value cut off of 0.05 as a screening tool for possibly meaningful associations. We discuss results as "significant" if the associated p-values are less than 0.05, but acknowledge that some results may be due to chance.

If the global deviance test indicates that the map is unlikely to be flat, we next want to locate areas of the map that exhibit unusually high or low disease odds. We examine pointwise departures from the null hypothesis of a flat surface using permutation tests. We obtained a distribution of the log odds at every point using the same set of permutations we used for calculating the global statistics. We defined areas of significantly decreased odds ("cold spots") to include all points that ranked in the lower 2.5% of the pointwise permutation distributions and areas of elevated odds ("hot spots") to include all points that ranked in the upper 2.5% of the pointwise permutation distributions. See Webster et al. [20] for a detailed description of the statistical methods.

In addition to hypothesis testing, we computed variability bands – a nonparametric relative of confidence intervals – to examine the precision of the point estimates for the models with a univariate smooth. We bootstrapped by resampling our data 1000 times, recomputing the log odds at each point on the grid [36]. We constructed the distribution of log odds at each grid point and recorded the log odds corresponding to the 2.5% and 97.5% percentiles. Since smoothing involves a tradeoff between bias and variance, variability bands do not have quite the same interpretation as confidence intervals [37], but they do indicate the precision of the point estimate.

In the one-dimensional time-only analyses, we constructed two separate univariate smooth models to examine the association between breast cancer risk during 1983–1993 and (1) earliest year lived in the study area or (2) residency duration. In the two-dimensional time-only analysis examining earliest calendar year and duration, we created a grid using all possible pairs of earliest year lived in the study area (1947–1993) and duration (1–47). Duration was calculated by subtracting the earliest year from the diagnosis or index year. We used a bivariate smooth to estimate the adjusted log odds at each cell on the grid.

In the space-only analysis, we created a rectangular grid covering the study area using the minimum and maximum longitude and latitude coordinates from the original data set as its dimensions. We clipped grid points lying outside the outline map of the study area or in areas where people cannot live (e.g., conservation areas). We used the spatial model to estimate the adjusted log odds at each grid point on the study area map.

Results from the generalized additive models were exported from S-plus [38] into ArcGIS [39] for mapping. In order to make visually comparable, we mapped all results using the same dark blue to dark red continuous color scale and same range of odds ratios, 0.25–2.50. The latter range covers most ORs observed in our analyses and prevents the color maps from being washed out by areas of extremely high ORs.

We analyzed the data in fixed-year time spans to study combined space-time effects of location and calendar years on breast cancer risk during 1983–1993. By dividing the data into datasets of overlapping time spans, we essentially smoothed over time. We used the optimal bandwidth from the smooth term in the one-dimensional calendar year analysis (earliest year a participant lived in the study area) to determine the time span for the data subsets. For each data subset, a spatially-smoothed map was created using methods similar to those in our space-only analysis, i.e., where the smooth term in the GAM is the coordinates for location and the span size is the same. We used the optimal span for the smooth term of location in the GAM for the first dataset in the models for the other data subsets to ensure that any differences observed in the maps were not due to differences in the span size. The combination of time spans and GAMs resulted in the simultaneous smoothing of space and calendar year.

These maps depict the risk of being diagnosed with breast cancer during 1983–1993 according to the location of participant residences in upper Cape Cod during historical time periods. They do not show the incidence of breast cancer during the historical periods. The maps were used to create a movie showing how breast cancer risk during 1983–1993 varied as historical residences changed over space and time. Maps were saved as image files and used to create a storyboard in Windows Movie Maker [40]. Each map plays for 0.5 seconds before transitioning to the next map.

## Abbreviations

AIC: Akaike's Information Criterion; GAM: generalized additive model; GIS: geographical information systems; ORs: odds ratios

## Competing interests

The authors declare that they have no competing interests.

## Authors' contributions

VV conducted the spatial-temporal analyses, drafted the manuscript, and provide analytical and editorial support. TW collaborated on all analytical and editorial decisions. JW provided statistical support and consulted on analytical and editorial issues. AA provided the data and assisted in epidemiologic analysis and editing. All authors read and approved the final manuscript.

## Supplementary Material

Additional file 1 Spatial-temporal analysis of breast cancer risk during 1983–1993. A movie that shows changing patterns of breast cancer risk during 1983–1993 based on participants' historical residence Click here for file
